# Hedonic tone is associated with left supero-lateral medial forebrain bundle microstructure

**DOI:** 10.1017/S0033291714001949

**Published:** 2014-08-15

**Authors:** T. Bracht, A. N. Doidge, P. A. Keedwell, D. K. Jones

**Affiliations:** 1 Cardiff University Brain Research Imaging Centre (CUBRIC), School of Psychology, Cardiff University, Cardiff, UK; 2 Neuroscience, Mental Health Research Institute, Cardiff University, Cardiff, UK

**Keywords:** Anhedonia, depression, diffusion tensor imaging, fibre tracking, remission, white matter

## Abstract

**Background.:**

The medial forebrain bundle (MFB) is an important pathway of the reward system. Two branches have been described using diffusion magnetic resonance imaging (MRI)-based tractography: the infero-medial MFB (imMFB) and the supero-lateral MFB (slMFB). Previous studies point to white-matter microstructural alterations of the slMFB in major depressive disorder (MDD) during acute episodes. To extend this finding, this study investigates whether white-matter microstructure is also altered in MDD patients that are in remission. Further, we explore associations between diffusion MRI-based metrics of white-matter microstructure of imMFB, slMFB and hedonic tone, the ability to derive pleasure.

**Method.:**

Eighteen remitted depressed (RD) and 22 never depressed (ND) participants underwent high angular resolution diffusion-weighted imaging (HARDI) scans. To reconstruct the two pathways of the MFB (imMFB and slMFB) we used the damped Richardson–Lucy (dRL) algorithm. Mean fractional anisotropy (FA) was sampled along the tracts.

**Results.:**

Mean FA of imMFB, slMFB and a comparison tract (the middle cerebellar peduncle) did not differ between ND and RD participants. Hedonic capacity correlated negatively with mean FA of the left slMFB, explaining 21% of the variance.

**Conclusions.:**

Diffusion MRI-based metrics of white-matter microstructure of the MFB in RD do not differ from ND. Hedonic capacity is associated with altered white-matter microstructure of the slMFB.

## Introduction

The medial forebrain bundle (MFB) is the central pathway of the reward system, which mediates feelings and expectations of pleasure (Schultz *et al.* 1997; Coenen *et al.* 2011). Traditionally the MFB was described as an assembly of loosely arranged, thin fibres extending from the septal area. Fibres traverse the lateral preoptico-hypothalamic area and proceed to the tegmentum of the midbrain (Nieuwenhuys *et al.* 2008). Coenen *et al.* (2009) were the first to reconstruct the MFB using diffusion magnetic resonance imaging (MRI)-based fibre tracking. In addition to this infero-medial MFB (imMFB) branch, the researchers described a supero-lateral branch (slMFB) proceeding from the ventral tegmental area (VTA) to the forebrain and the frontal lobe (Coenen *et al.* 2009, 2012).

Anhedonia, the reduced capacity to derive pleasure from previously rewarding experiences, is a core feature of major depressive disorder (MDD). Given its prominent role in the reward system (Schultz *et al.* 1997; Nestler & Carlezon, 2006), the MFB has become a major focus in the search for the neurobiological underpinnings of MDD (Blood *et al.* 2010; Bracht *et al.* 2014). In particular, the slMFB may be involved in the neurobiology of depression (Schlaepfer *et al.* 2014) and becomes an increasingly important region for deep brain stimulation (DBS) in treatment-resistant MDD (Schlaepfer *et al.* 2013).

Diffusion MRI allows white-matter microstructure to be probed by indirectly measuring the hindrance of diffusion of water molecules (Basser *et al.* 1994). The most commonly used diffusion MRI-based measure in clinical studies is fractional anisotropy (FA) (Basser & Pierpaoli, 1996). Reductions in FA indicates differences in barriers to diffusion of water molecules. This may reflect altered white-matter microstructure, which in turn could have functional significance in the mediation of hedonic responses to positive events (Keedwell *et al.* 2012).

Two studies have used diffusion MRI in order to specifically assess white-matter microstructure of the MFB. One study demonstrated a trend towards reduced FA in the imMFB in currently depressed patients (Blood *et al.* 2010). A recent diffusion MRI-based fibre-tracking approach identified reduced FA in severely depressed melancholic MDD patients in segments of the slMFB connecting the VTA with the medial orbitofrontal cortex (OFC) and the dorsolateral prefrontal cortex (dlPFC) (Bracht *et al.* 2014). Lower FA was associated with more pronounced anhedonia and depression severity (Bracht *et al.* 2014). Moreover, voxel-based diffusion MRI (Zou *et al.* 2008; Liao *et al.* 2013), region of interest (Bae *et al.* 2006; Blood *et al.* 2010) and tract-based spatial statistics (TBSS) (Korgaonkar *et al.* 2011; Zhu *et al.* 2011) studies have demonstrated reductions of FA in acute depression in the anterior limb of the internal capsule and in prefrontal brain regions that likely incorporate segments of the slMFB (Coenen *et al.* 2009, 2012).

However, it has not been determined if white-matter changes in these reward tracts are state-dependent or trait markers of vulnerability to depression. To date, no studies have examined if changes in FA in the MFB persist into remission.

Decreases of FA in the ventromedial prefrontal cortex, a region adjacent to the slMFB, were found in treatment-resistant depressed MDD but not in remitted depressed (RD) (de Diego-Adelino *et al.* 2014), suggesting that remodelling of white-matter microstructure occurs during remission. However, we note previous studies where voxel-based analyses failed to show a group effect, but tract-specific approaches showed a significant effect (Cullen *et al.* 2010; Keedwell *et al.* 2012; Bracht *et al.* 2014).

Based on previous work, the present study was designed to test the following hypotheses: First, that FA would be reduced in the MFB in RD compared with never depressed (ND) individuals, consistent with the proposition that this represents a trait marker of MDD. Second, that, consistent with findings in acute MDD, FA in the slMFB tract would correlate positively with a measure of hedonic tone (or higher FA = lower anhedonia).

In accordance with previous approaches dividing tracts into subdivisions (Jones *et al.* 2013*a*) we reconstructed the two branches of the MFB (imMFB, slMFB) and analysed them separately. As a methodological refinement of previous studies, we employed the damped Richardson–Lucy (dRL) algorithm (Dell'acqua *et al.* 2010), which in contrast to diffusion tensor imaging (DTI) estimates multiple directions within a single voxel, and is therefore capable of improving the accuracy of tract reconstruction through regions of complex fibre architecture. Due to the particular importance of the VTA for the experience of pleasure (Schultz *et al.* 1997; Nestler & Carlezon, 2006), we included dorsal segments of the VTA projecting to the nucleus accumbens (NAc) and the prefrontal cortex (Nieuwenhuys *et al.* 2008; Bracht *et al.* 2014) in our tract reconstructions.

To establish the specificity of potential findings we reconstructed the middle cerebellar peduncle (MCP) as a comparison tract. We also performed a whole brain group comparison of FA to complement the tract reconstruction approach.

## Method

### Sample and measures

Eighteen RD, unmedicated women with a history of MDD and 22 healthy controls without a history of MDD (ND, never depressed) were recruited from the staff and student body of the School of Psychology, Cardiff. We recruited individuals of one gender only to reduce the potential effect of gender-based variability of brain structure (Kanaan *et al.* 2014), thereby increasing the power to detect group differences. Females were specifically chosen because they have a higher incidence of depression than men, attributable to a greater incidence of first onset as opposed to chronicity or recurrence (Kessler *et al.* 1993). Controls were matched for age, gender and pre-morbid intelligence. Inclusion criteria for all participants were right handedness and fluency in English. Exclusion criteria were contraindications for magnetic resonance imaging (MRI) scans, a diagnosis of Axis I disorder, a current episode of depression, substance dependence and psychotropic medication. The Mini International Neuropsychiatric Inventory (MINI; Sheehan *et al.* 1998) was used to exclude a current episode of depression in all participants. Further, the MINI was used to confirm a history of a depressive episode in RD and the absence of a history of depression in ND (see Appendix). Results of the MINI were corroborated by a medical history. The MINI was also used to screen participants for a history of psychiatric disorders and drug or alcohol dependence. We employed additional questions (regarding hospitalization, treatments, suicidal behaviour and psychosis) in order to rate RD participants on the Bipolar Affective Disorder Dimension Scale (BADDS) – a dimensional scale for rating lifetime psychopathology in bipolar and unipolar disorders, taking in to account the number and severity of episodes (Craddock *et al.* 2004). All participants completed the Beck Depression Inventory (BDI-II; Beck *et al.* 1996), the Fawcett Clark Pleasure Scale (FCPS; Fawcett *et al.* 1983) for assessment of hedonic tone and the National Adult Reading Test (NART; Nelson & Willison, 1991), an assessment of pre-morbid intelligence. The cut-off score for moderate depression according to the BDI-II is 14. Higher scores on the FCPS indicate more pronounced capacity to derive pleasure. All questionnaires were completed in the presence of a psychologist who ensured that questionnaires were completed correctly and to ensure that no misunderstandings occurred. All participants provided written informed consent. The study was approved by the School of Psychology Research Ethics Committee.

### Diffusion MRI procedure

#### Diffusion MRI scanning

Diffusion-weighted MRI data were acquired on a 3 T GE Signa HDx system (General Electric Healthcare, UK) using a peripherally gated twice-refocused pulse-gradient spin-echo echo-planar imaging sequence providing whole oblique axial (parallel to the commissural plane) brain coverage. Data were acquired from 60 slices of 2.4 mm thickness, with a field of view 23 cm, and an acquisition matrix of 96 × 96 (yielding isotropic voxels of 2.4 × 2.4 × 2.4 mm, reconstructed to a resolution of 1.9 × 1.9 × 2.4 mm). Echo time (TE) was 87 ms and parallel imaging (ASSET factor × 2) was used. Diffusion-encoding gradients (*b* × 1200 s/mm^2^) were applied along 60 isotropically distributed directions (Jones *et al.* 1999). Six additional non-diffusion-weighted scans were collected. The acquisition time was approximately 26 min.

#### Structural MRI scanning

T1-weighted structural scans were acquired using an oblique axial, 3D fast-spoiled gradient recalled sequence (FSPGR) with the following parameters: TR = 7.9 ms; TE = 3.0 ms, inversion time = 450 ms, flip angle = 20°, 1 mm isotropic resolution, with total acquisition time of ~7 min.

#### Diffusion MRI data pre-processing

The data were corrected for distortions and subject motion using an affine registration to the non-diffusion-weighted images, with appropriate re-orienting of the encoding vectors (Leemans & Jones, 2009). A single diffusion tensor model was fitted (Basser *et al.* 1994) to the data in order to compute quantitative parameters such as FA. The dRL algorithm was used to estimate the fibre orientation density function (fODF) in each voxel (Dell'acqua *et al.* 2010). Following the method of Pasternak *et al.* (Pasternak *et al.* 2009; Metzler-Baddeley *et al.* 2012), a correction for free water contamination of the diffusion tensor based estimates was applied, before sampling diffusion properties (e.g. FA) along the tracts.

### Tractography

Deterministic tractography was performed using ExploreDTI (Leemans *et al.* 2009) following peaks in the fODF reconstructed from the dRL algorithm (Dell'acqua *et al.* 2010; Jeurissen *et al.* 2013). For each voxel in the dataset, streamlines were initiated along any peak in the fODF that exceeded an amplitude of 0.05. Thus (in contrast to DTI-based methods), multiple fibre pathways could be generated from any voxel. Each streamline continued in 0.5 mm steps following the peak in the ODF that subtended the smallest angle to the incoming trajectory. The termination criterion was an angle threshold >45°.

#### Tract reconstruction

**Fig. 1.**
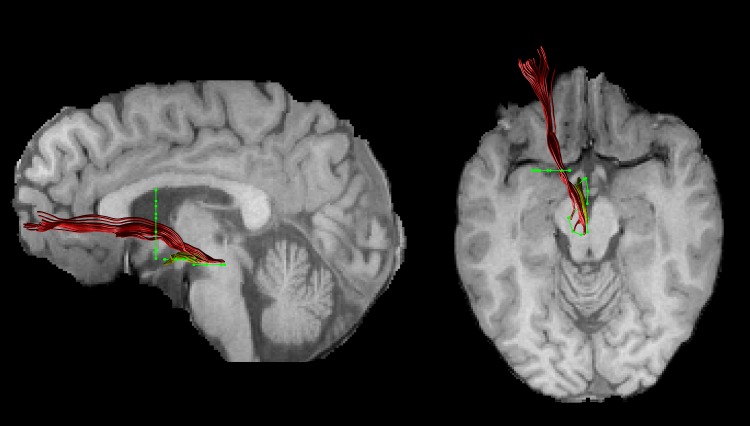
An individual example of the two reconstructed branches is shown for the left imMFB (yellow) and the left slMFB (red). Regions of interest are displayed in green.

The FA images of each subject were warped to their respective FSPGR image using the linear registration tool FLIRT (Jenkinson *et al.* 2002). Inverse parameters were applied to transform the FSPGR image to the FA image. Afterwards, FSPGR images were used as a template to draw regions of interest (ROI) for virtual dissection of the different branches of the MFB. Seed regions were drawn by one experimenter (T.B.) who was blind to the diagnosis of participants. For both the imMFB and slMFB a ROI surrounding the VTA was drawn in the horizontal section. Anatomical borders were laterally the substantia nigra, anteriorly the mammillary bodies and posteriorly the red nucleus. For reconstruction of the imMFB a second ROI surrounding the hypothalamus was drawn on a horizontal section one section above the VTA ROI. For reconstruction of the slMFB a second ROI was drawn surrounding caudate and putamen on a coronal section at the height of the NAc. The anatomical course of each tract was carefully checked for each subject (see Fig. 1). Due to the particular interest in the role of the MFB in reward processing, the focus was placed on segments of the MFB dorsal to the VTA including projections from the VTA to NAc, hypothalamus and the OFC, core regions of reward processing (Haber & Knutson, 2010). Seed regions for the comparison tract (MCP) were drawn on a coronal section, where left and right MCP can be clearly identified. The MCP was chosen because it can be reliably isolated but is not predicted *a priori* to be affected in RD. Because of the spatial overlap of left and right MCP in regions of the pontine nuclei (Nieuwenhuys *et al.* 2008), the MCP was treated as a sole ROI. Mean FA was derived for each reconstructed tract for each subject. In addition, the average mean diffusion (MD) and the axial and radial diffusivity (AD, RaD) were computed, to facilitate follow-up of any group differences seen in FA, our primary outcome measure.

### Statistical analysis

Statistical analyses were performed using SPSS (SPSS Inc., USA). A MANCOVA was used to explore main effects of group (ND *v.* RD) and hedonic tone (FCPS score), and their interactions on mean FA of the four respective tracts. To follow-up any significant main effects of group, hedonic tone or group × hedonic tone interactions, four separate ANCOVAs were calculated [one ANCOVA for each tract, fixed factor group (ND, RD), covariate hedonic tone]. The *p* value was adjusted using a Bonferroni correction for multiple comparisons (0.05/4 = 0.0125). Where significant effects on mean FA were found, analyses of the effects on additional metrics (MD, RaD, AD) were explored.

#### Whole brain voxel-wise analysis

Voxel-wise statistical analysis of FA data was performed using FSL TBSS software (Smith *et al.* 2004, 2006). FA data were projected onto a mean FA tract skeleton, before applying voxel-wise cross-subjects statistics. The tract skeleton was thinned using an FA threshold >0.2. Group comparisons between RD and ND of FA on this fibre skeleton were then performed using threshold-free cluster-enhancement (TFCE). Group comparisons were deemed to be significant at a cluster threshold of *p* < 0.05. Correlations between FCPS score and FA across the skeleton were also examined.

## Results

### Sample characteristics

Groups did not differ regarding age, gender, pre-morbid intelligence, hedonic tone (FCPS score) or handedness. None of the participants met criteria for MDD according to the MINI. RD patients had significantly higher BDI scores (for details see Table 1).

**Table 1.** Demographics for never depressed and remitted depressed participants

	RD (*n* = 18)	ND (*n* = 22)	*p*
Age (years)	22.4±3.6	22.5±4.5	0.933
Female gender (%)	100	100	
Right handedness (%)	100	100	
Premorbid intelligence	113±5	112±5	0.647
Fawcett score	120±11	122±11	0.687
BDI score	11.4±10.4	2.7±4	0.001*
Number of episodes	2.22±3.6	0	<0.001*

RD, Remitted depressed; ND, never depressed; BDI, Beck Depression Inventory.

* Significant at *p* < 0.05.

Our participants had a mean score of 65 ± 10 on the BADDS, indicating a moderate to severe history of depression. Seven RD participants had a history of treatment with antidepressive medication, while 11 were medication-naive, and four had a history of treatment with psychotherapy. None had a history of psychotic depression or had been hospitalized for treatment. Fifteen patients had a history of depressive episodes that met DSM-IV criteria for melancholic depression, as defined by the MINI and four had a history of a suicide attempt.

### Tract-specific measurements

The MANCOVA revealed a main effect of hedonic tone (FCPS score) on mean FA across the four tracts (*F*_4,33_ = 4.112, *p* = 0.008), but no main effect of group (*F*_4,33_ = 0.522, *p* = 0.720) or significant group × hedonic tone interaction (*F*_4,33_ = 0.454, *p* = 0.769). This main effect was followed up using four separate ANCOVAs. There was only a significant main effect of hedonic tone on mean FA for the left slMFB (*F*_1,36_ = 10.712, *p* = 0.002), but not for the left imMFB (*F*_1,36_ = 1.812, *p* = 0.185), right imMFB (*F*_1,36_ = 2.501, *p* = 0.344) or right slMFB (*F*_1,36_ = 0.920, *p* = 0.344).

**Fig. 2.**
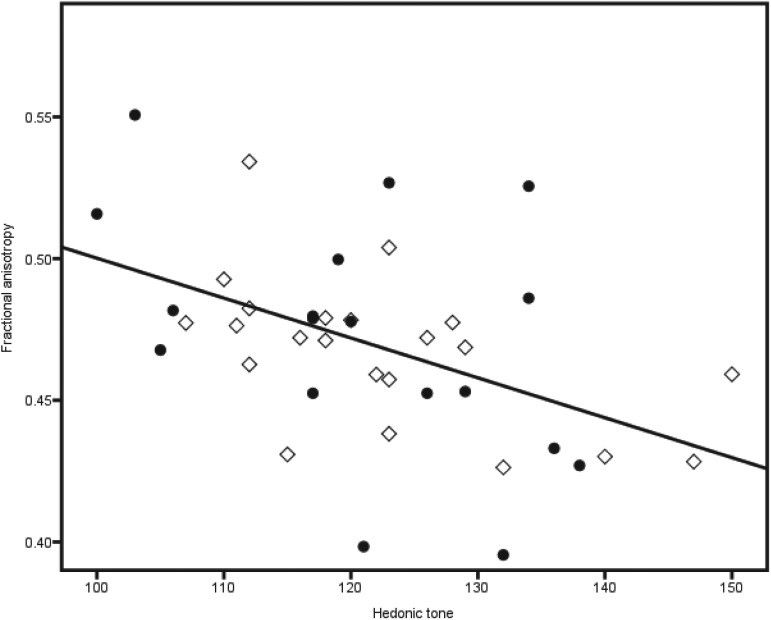
The negative correlation across the whole sample between mean fractional anisotropy of the left slMFB and hedonic tone is displayed. ◇, Never depressed; ●, remitted depressed.

In accordance with these findings there was a sole negative correlation between FCPS scores and mean FA of the left slMFB (*r* = −0.48, *p* = 0.002) across all individuals (Fig. 2), explaining 20.6% of the variance. FA of the right slMFB (*r* = −0.146, *p* = 0.369), left imMFB (*r* = −0.232, *p* = 0.150) and right imMFB (*r* = −0.247, *p* = 0.125) did not correlate with FCPS scores. Mean FA of none of the tracts correlated with BDI scores.

Secondary correlational analyses with diffusion properties demonstrated that the negative correlation between FCPS scores and FA mainly reflected changes in RaD (RaD: *r* = 0.460, *p* = 0.003; MD: *r* = 0.337, *p* = 0.033; AD: *r* = −0.281, *p* = 0.079).

FA in the comparison tract (MCP) did not differ between groups (mean FA RD = 0.46 ± 0.03, mean FA ND = 0.47 ± 0.03, *T* = 1.215, df = 38, *p* = 0.236). There was no significant correlation between FCPS and MCP FA (*r* = −0.32, *p* = 0.842).

### Voxel-wise whole brain analysis

For the TBSS results there were no significant group differences in FA and no significant correlations between FCPS scores and FA in any brain region.

## Discussion

Our study has two main findings. First, we found no differences in FA for any MFB region between unmedicated RD and ND individuals, suggesting that microstructural abnormalities of the MFB are not present in individuals with remitted depression. Second, we have demonstrated a negative correlation between the capacity to derive pleasure and mean FA of the left slMFB in all individuals, irrespective of depression history. Mean FA explained 21% of the variance of hedonic tone. Decreases of FA were mainly driven by decreases of RaD. Hedonic tone did not correlate with FA in the control tract or the imMFB.

The absence of group differences in FA in our study leads us to reject our first hypothesis. However, this complements findings of reduced FA of the slMFB in severely melancholic but not in moderately depressed patients (Bracht *et al.* 2014). Similarly to another study, whole brain FA reductions were observed in chronic treatment-resistant patients but not in remitted, unmedicated patients (de Diego-Adelino *et al.* 2014). Therefore, while previous research points to white-matter microstructure alterations of the slMFB in severely depressed patients (Bracht *et al.* 2014), to date there is no evidence for altered structural connectivity in remission.

Collectively, these results suggest that reductions in FA in the MFB are state-dependent effects and not trait markers of vulnerability, and only appear in melancholic depression. It follows that neuroplastic changes could occur upon recovery, reversing changes observed during the acute illness. White-matter microstructure may change even within very short time-scales (Sagi *et al.* 2012), including after moderate interventions such as learning how to juggle (Scholz *et al.* 2009) or half an hour of aerobic exercise per day (Erickson *et al.* 2011). Consistent with this explanation a 1-year follow-up longitudinal study in late-life depression demonstrated normalization of FA in other white-matter tracts upon recovery (Taylor *et al.* 2011). Furthermore, FA of limbic pathways may differ between treatment responders and non-responders (Taylor *et al.* 2008; Delorenzo *et al.* 2013), which is also suggestive of white-matter remodelling during recovery. Longitudinal studies of changes in MFB FA in response to treatment are indicated to further explore the neuroplasticity of these tracts in relation to recovery.

A further explanation is that, while some of the RD individuals in this study might go on to develop a more severe or treatment-resistant course, any abnormalities of white-matter microstructure in this group could be masked by those RD individuals with a putatively better prognosis. Longitudinal studies would also inform this research question.

Our results suggest that lower FA in the left slMFB is associated with more pronounced capacity to derive pleasure in RD and ND. Hence, the correlation is in the opposite direction to that hypothesized, and previously demonstrated in acute depression, where slMFB FA correlated negatively with anhedonia scores (Bracht *et al.* 2014).

However, different microstructural changes could be occurring in the different populations while still having similar effects on hedonic processing. For example, greater myelination and larger axonal diameter both increase conduction velocity in a tract but have opposite effects on FA, all other factors being constant. Therefore, changes in FA alone cannot define any particular change in ‘fibre integrity’ (Jones *et al.* 2013*b*). Novel white-matter mapping techniques such as the composite hindered and restricted model of diffusion (CHARMED; De Santis *et al.* in press) or multicomponent-driven equilibrium pulse observation of T1 and T2 (McDESPOT; Deoni *et al.* 2005) provide subcompartment-specific measures (e.g. on axonal diameter or myelination) and could lead to a better understanding of the neurobiological underpinnings of these findings.

The identified associations between individual differences in the white-matter microstructure of the slMFB and the capacity to derive pleasure are indirectly supported by animal research and by functional MRI (fMRI) and positron emission tomography (PET) studies in humans. Research in animals convincingly demonstrates a key role of the VTA, NAc and OFC in reward processing (Schultz *et al.* 1997; Haber & Knutson, 2010). Furthermore, fMRI and PET studies in humans demonstrate activations of NAc, VTA and OFC when perceiving pleasure (Drevets *et al.* 2001; Kringelbach, 2005). Individuals with more pronounced hedonic responses experience relatively greater activations in these areas to the same pleasurable stimulus (Breiter *et al.* 1997; Blood & Zatorre, 2001; O'Doherty *et al.* 2001). This also appears to be true in depression, although the evidence in MDD is less consistent (Keedwell *et al.* 2005; Smoski *et al.* 2009; Zhang *et al.* 2013). The slMFB structurally connects these core regions of the reward system (Nieuwenhuys *et al.* 2008), and may therefore play an essential role in integrating information leading to the perception of pleasure. Therefore, our finding of an association between hedonic tone and microstructure of the slMFB is consistent with this body of literature. However, one obvious caveat is that our study design does not allow us to establish the direction of causality.

Pleasurable experiences are derived from natural rewards such as food and sex and from social interactions (Nestler & Carlezon, 2006). The slMFB provides an essential link to the prefrontal cortex, which interprets the rewarding potential of external cues based on past experience, and therefore contributes to motivated and goal-directed behavior (Haber & Knutson, 2010). Moreover, patent slMFB connections are essential for achieving a balance between reward and the panic/grief systems (Coenen *et al.* 2012).

MFB microstructure could mediate individual differences in both subclinical (trait) anhedonia, as in this study, and clinical (depressive) anhedonia. The central importance of MFB function in depression is supported by DBS research: DBS targeting the ventral striatum/MFB provides some relief of depression in a subset of treatment-resistant patients (Malone *et al.* 2009; Bewernick *et al.* 2010; Schlaepfer *et al.* 2013).

The lack of any significant findings for our TBSS analyses is consistent with increasing evidence (Kanaan *et al.* 2006; Keedwell *et al.* 2012) that tract-averaging approaches are more sensitive than voxel-based approaches; possibly because subtle microstructural differences only reach significance if averaged over the whole tract, but not if compared on a voxel by voxel basis.

This study has some limitations. First, although none of the RD participants met criteria for diagnoses of a current episode for depression, groups differed with regard to depressive symptomatology. However, scores on the BDI-II did not correlate with FA of imMFB and slMFB which may be as a result of small variance in BDI scores. Moreover, groups did not differ regarding hedonic tone, and there was no group × hedonic tone interaction. Second, our young sample, who remained well while unmedicated, with relatively few previous episodes, may not be representative of the majority of patients with MDD seen in clinical practice. However, including medicated individuals would have made any results difficult to interpret. Future studies could include older patients, while attempting to control for the independent effect of age on white-matter microstructure *per se*. Third, since we aimed to investigate remitted, fully recovered participants our participants did not receive ongoing treatment. Therefore we did not have access to clinical files for validation of previous diagnoses. Fourth, we did not have information on the menstrual cycle of participants which may influence white-matter microstructure (De Bondt *et al*. 2013).

In conclusion, this is the first tractography study to link the capacity to derive pleasure to white-matter microstructure of specific subcompartments of the MFB. We found a negative association between hedonic tone and mean FA of the slMFB in a non-clinical group. Our findings corroborate the important role of the slMFB in reward processing and its potential role in depression. Longitudinal studies are needed to assess the prognostic value of slMFB microstructure in MDD, and to investigate if white-matter changes occur in tandem with clinical recovery. Finally, advanced white-matter mapping techniques such as CHARMED (De Santis *et al.* in press) or McDESPOT (Deoni *et al.* 2005) provide promise in clarifying the microstructural changes that underlie changes in FA.

## Appendix

**Table:**

**HISTORY OF DEPRESSION QUESTIONNAIRE A1–A4 MAJOR DEPRESSION**			
(means: go to the diagnostic boxes, circle NO in all diagnostic boxes, and move to the next module)			
A1	Have you ever been consistently depressed or down, most of the day, nearly every day, for at least 2 weeks?	NO	YES
A2	During your lifetime, have you experienced a period of 2 weeks or more when you have been less interested in most things or less able to enjoy the things you used to enjoy most of the time?	NO	YES*
	is **A1** or **A2** coded YES?	NO	YES
A3	**Over the two weeks, when you felt depressed or uninterested:**		
a	Was your appetite decreased or increased nearly every day? Did your weight decrease or increase without trying intentionally (i.e. by ± 5% of body weight or ± 8 lb or ± 3.5 kg, for a 160 lb/70 kg person in a month)?	NO	YES*
	if YES to either, code YES.		
b	Did you have trouble sleeping nearly every night (difficulty falling asleep, waking up in the middle of the night, early morning wakening or sleeping excessively)?	NO	YES
c	Did you talk or move more slowly than normal or were you fidgety, restless or having trouble sitting still almost every day?	NO	YES*
d	Did you feel tired or without energy almost every day?	NO	YES
e	Did you feel worthless or guilty almost every day?	NO	YES
f	Did you have difficulty concentrating or making decisions almost every day?	NO	YES
g	Did you repeatedly consider hurting yourself, feel suicidal, or wish that you were dead?	NO	YES
h	Loss of confidence or self-esteem	**NO**	**YES***
	are 5 or more answers (**A1**–**A3**) coded **YES?**	***MAJOR*** ***DEPRESSIVE*** ***EPISODE***	
	if patient has current major depressive episode continue to A4, otherwise move to module b:		
A4			
a	During your lifetime, did you have other periods of two weeks or more when you felt depressed or uninterested in most things, and had most of the problems we just talked about?	NO	YES
b	Did you ever have an interval of at least 2 months without any depression and any loss of interest between 2 episodes of depression?	**NO**	**YES**
		***RECURRENT*** ***DEPRESSIVE*** ***DISORDER***	

* If patient has major depressive episode, current, code **YES** in corresponding questions on page 5.
